# Blaming automated vehicles in difficult situations

**DOI:** 10.1016/j.isci.2021.102252

**Published:** 2021-03-01

**Authors:** Matija Franklin, Edmond Awad, David Lagnado

**Affiliations:** 1 Department of Experimental Psychology, University College London, London WC1E 6BT, UK; 2 Department of Economics, University of Exeter Business School, Exeter EX4 4PU, UK

**Keywords:** Artificial Intelligence, Psychology, Research Methodology Social Sciences

## Abstract

Automated vehicles (AVs) have made huge strides toward large-scale deployment. Despite this progress, AVs continue to make mistakes, some resulting in death. Although some mistakes are avoidable, others are hard to avoid even by highly skilled drivers. As these mistakes continue to shape attitudes toward AVs, we need to understand whether people differentiate between them. We ask the following two questions. When an AV makes a mistake, does the perceived difficulty or novelty of the situation predict blame attributed to it? How does that blame attribution compare to a human driving a car? Through two studies, we find that the amount of blame people attribute to AVs and human drivers is sensitive to situation difficulty. However, while some situations could be more difficult for AVs and others for human drivers, people blamed AVs more, regardless. Our results provide novel insights in understanding psychological barriers influencing the public's view of AVs.

## Highlights

• Attributed blame to machine and human drivers is sensitive to situation difficulty
• Mistakes in simple situations receive more blame than in novel or complex situations
• Machine drivers receive more blame, across different situations

## Graphical abstract

**Figure:**
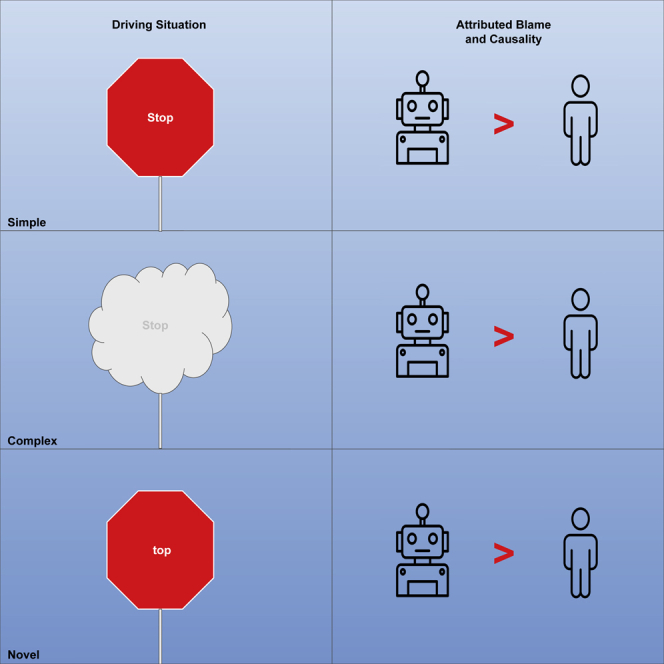

## Introduction

Once properly prepared and finalized to deploy on the roads, automated vehicles (AVs) are expected to bring many benefits, such as decreasing the rate of car crashes (Gao et al., 2014), reducing pollution (Spieser et al., 2014), and increasing traffic efficiency (van Arem et al., 2006). Assuming that AVs will overcome all remaining technical challenges before they are ready to deliver these benefits, while exhibiting no serious drawbacks, their deployment on a larger scale would be beneficial. However, these benefits will not be realized if people are not ready to buy them, and various considerations contribute to the public's aversion to adopting this technology.

Understanding people's attitudes is key to identifying these considerations, and working to address any potential concerns (Shariff et al., 2017; Schlögl et al., 2019; Sun and Medaglia, 2019; Bonnefon et al., 2020; Dellaert et al., 2020). The public's views and trust toward AVs is a major factor that predicts adoption of autonomous vehicles (Lee and Moray, 1992, 1994; Gefen et al., 2003; Carter and Bélanger, 2005). Evidence suggests that people require AVs to be multiple orders of magnitude safer than human drivers (Liu et al., 2019). As argued in (Awad et al., 2020), negative public reaction may result in inflated prices of this technology (Geistfeld, 2017) and may shape how a tort-based regulatory scheme would turn out, both of which can influence the rate of adoption.

In such cases of high stakes (safety of life), human attitude is mainly shaped by situations of failure. An autonomous vehicle may navigate its way successfully on the roads for long periods of time but will still be slammed for failing to avoid a crash in one situation. This asymmetric effect of performance on the public's attitude is amplified by the wide coverage of the few crashes by AVs, compared to the coverage of successful performance or achieved milestones by these AVs, and also compared to crashes by human drivers (Lambert, 2018). The strong reactions these few crashes have elicited point to the importance of focusing on mistakes and the failure situations to understand the publics' attitude.

Understanding how we react to mistakes by machines (as compared to those by humans) is not an easy task. There is strong evidence that people react differently to mistakes made by machines and humans (Dietvorst et al., 2014, 2015; Malle et al., 2015; Awad et al., 2020). There are also reasons to believe that people assign blame differently based on the difficulty of encountered situations. Complicating matters, perceived difficulty of the situation may vary depending on the agent behind the steering wheels. For example, a drunk person jumping in front of a car may be considered a difficult situation for a human driver but not for a machine driver (Goodall, 2016). Likewise, a novel situation in which the only way to overtake a stationary vehicle is to illegally cross the central line may be deemed more difficult for a machine driver than for a human driver.

In this study, we focus on two questions: (1) when an automated car makes a mistake, does the perceived difficulty or novelty of the situation predict blame attributed to it? (2) How does that blame attribution compare to a human driving a regular car?

**Figure 1:**
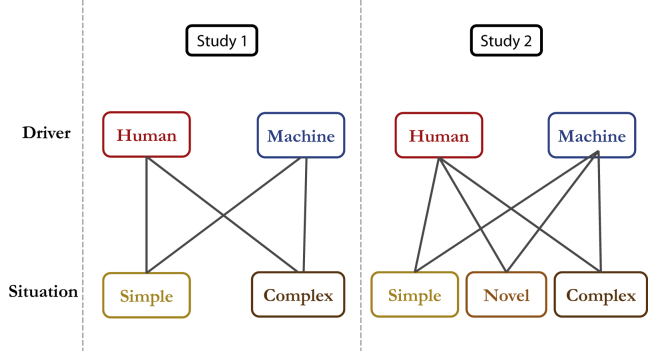
Experimental design of the two studies The edges connecting *Driver* and *Mistake* represent the experimental groups participants were allocated to, with a total of four experimental groups for study 1: human driver in a simple situation, human driver in a complex situation, machine driver in a simple situation and machine driver in a complex situation; and six experimental groups for study 2 – same as in study 1 with the addition of two new experimental groups: human driver in a novel situation and machine driver in a novel situation.

To answer these questions, we devise two studies that look at mistakes made by human drivers and machine drivers in driving situations that span different levels of difficulty (see Figure 1). Specifically, we consider three types of situations: (a) simple driving situations: those that most humans would consider easy to navigate without making mistakes (most of the time). (b) complex situations: those that add extra layers of difficulty or complication to the simple driving situation, requiring a higher level of competence to navigate while avoiding making mistakes, and (c) novel situations: those that are less likely to be encountered while driving (than simple or complex situations) and require novel inferences and actions that would not have been part of pre-training. The consideration of complex and novel situations here is crucial, given that what is deemed as difficult for a human driver (e.g., split-second decisions) can be an easy task for an AV. On the other hand, a novel situation (e.g., having to make an illegal move to overtake a stationary vehicle) could prove more challenging for an AV than for a human driver (Marcus and Davis, 2019).

## Results

**Figure 2:**
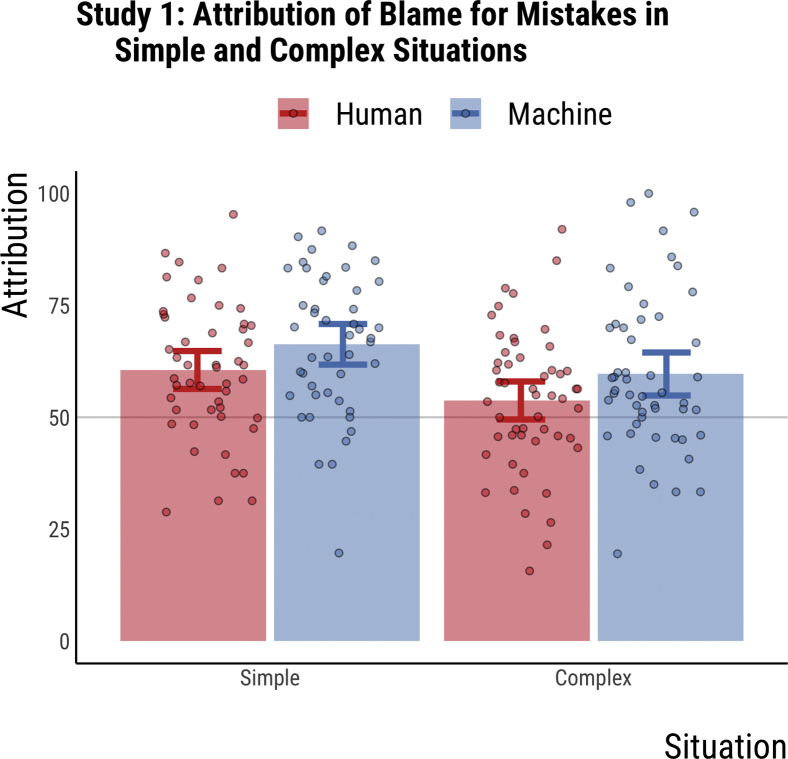
Attribution of blame for mistakes in simple and complex situations in study 1 Data from study 1 (n = 198). Participants were randomly allocated to one of four groups. The x axis represents the situation difficulty (simple vs. complex). The y axis represents blame attribution. Blue and red bars represent the mean blame attribution to the machine driver (AV) and the human driver, respectively. Error bars represent 95% confidence intervals of the mean. Each circle represents an individual's blame score (averaged over six stories). Machine drivers are blamed more than human drivers in total and across the two types of scenarios [F(196, 2) = 6.17, p = .014]. Machine and human drivers are blamed for making mistakes more in simple situations than in complex situations [F(196, 2) = 8.36, p = .004]. Data are represented as mean ± SEM.

In study 1, 198 participants were allocated randomly to one of four conditions: (driver: human vs. machines): x (situation: simple vs. complex). Each participant read six different stories of a mistake by a driver resulting in a crash, and then assigned scores of blame and causality to the driver. These scores were summed up into two separate blame and causality scores. The descriptive statistics and the individuals' blame scores are available in Figure 2 (causality scores are very similar).

Our results show that machines receive more blame and causality attribution than humans for either type of mistakes and that humans and machines are blamed more for mistakes in simple situations (see Figure 2). For blame attributions, results from a 2 × 2 ANOVA (driver x situation) show that machine drivers are blamed significantly more than human drivers for doing the same mistakes [F(196, 2) = 5.82, p = .011] and that all drivers get more blame for mistakes in simple, rather than complex, situations [F(196, 2) = 6.73, p = .004]. These results were replicated for causality attributions with machines receiving higher causality attributions [F(196, 2) = 6.18, p = .008] and drivers committing mistakes in simple situations being perceived as more causally responsible [F(196, 2) = 4.66, p = .045].

**Figure 3:**
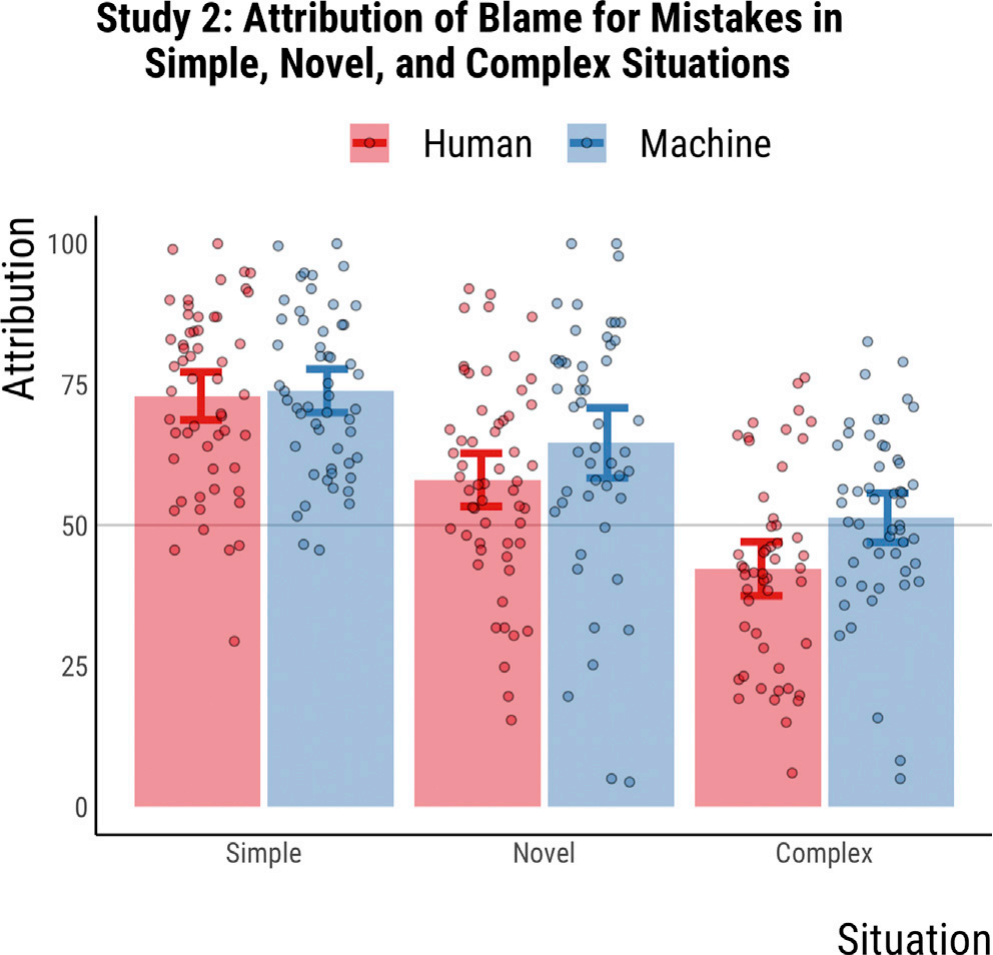
Attribution of blame for mistakes in simple, novel, and complex situations in study 2 Data from study 2 (n = 317). Participants were randomly allocated to one of six groups. The x axis represents the situation difficulty (simple vs. novel vs. complex). The y axis represents blame attribution. Blue and red bars represent the mean blame attribution to the machine driver (AV) and the human driver, respectively. Error bars represent 95% confidence intervals of the mean. Each circle represents an individual's blame score (averaged over five stories). Machine drivers are blamed more than human drivers in total and across two types of scenarios (inconclusive for simple situations) [F(315, 2) = 4.99, p = .026]. There were significant differences in blame across driving situations [F(314, 3) = 59.05, p < .001], with mean score for blame in simple situations being significantly higher than mean scores for blame in novel [differences in means = 61.26, 95% CI: [32.69, 89.84], p < .001] and complex situations [differences in means = 132.76, 95% CI: [103.98, 161.54], p < .001]. Mean scores for blame in novel situations were higher than those in complex situations [differences in means = 71.49, 95% CI: [42.58, 100.41], p < .001]. Data are represented as mean ± SEM.

In study 2, 317 participants were allocated randomly to one of six conditions: (driver: human vs. machines): x (situation: simple vs. novel vs. complex). Each participant read five different stories of a mistake by a driver resulting in a crash, and then assigned scores of blame and causality to the driver. Participants also rated the driving situation in terms of novelty and difficulty. Blame judgments and causality attributions were summed up into two separate blame and causality scores. The descriptive statistics for blame scores are available in Figure 3 (causality scores are very similar).

Our results show that machines receive more blame and causality attribution than humans for mistakes in novel and complex mistakes (see Figure 3). Furthermore, humans and machines are blamed the most for mistakes in simple situations, followed by novel and then complex situations. For blame attributions, results from a 2 × 3 ANOVA (driver x situation) show that overall machine drivers are blamed significantly more than human drivers for doing the same mistake [F(314,3) = 7.81, p = .006] and that all drivers receive significantly different levels of blame for mistakes in different driving situations [F(314,3) = 60.75, p < .001]. Post hoc comparisons using the Tukey honestly significant difference (HSD) test indicated that the mean blame score in simple situations (M = 367, SD = 75.98) was significantly higher than in novel (M = 305.74, SD = 101.98) and complex (M = 234.24, SD = 86.5) situations. Further, the mean blame score in novel situations was significantly higher than in complex situations. The patterns in this ANOVA were replicated in ANOVAs performed for individual items (See S2).

These results were replicated for causality attributions with machines receiving higher causality attributions [F(314,3) = 11.66, p < .001] and drivers rated as differentially causal for different scenarios [F(314,3) = 67.48, p < .001]. Post hoc comparisons using the Tukey HSD test indicated that the mean causality score in simple situations (M = 383.3, SD = 71.2) was significantly higher than in novel (M = 313.1, SD = 99.65) and complex (M = 251.38, SD = 79.87) situations. Further, the mean causality score in novel situations was significantly higher than in complex situations. The patterns in this ANOVA were replicated in ANOVAs performed for individual items (See S2).

**Figure 4:**
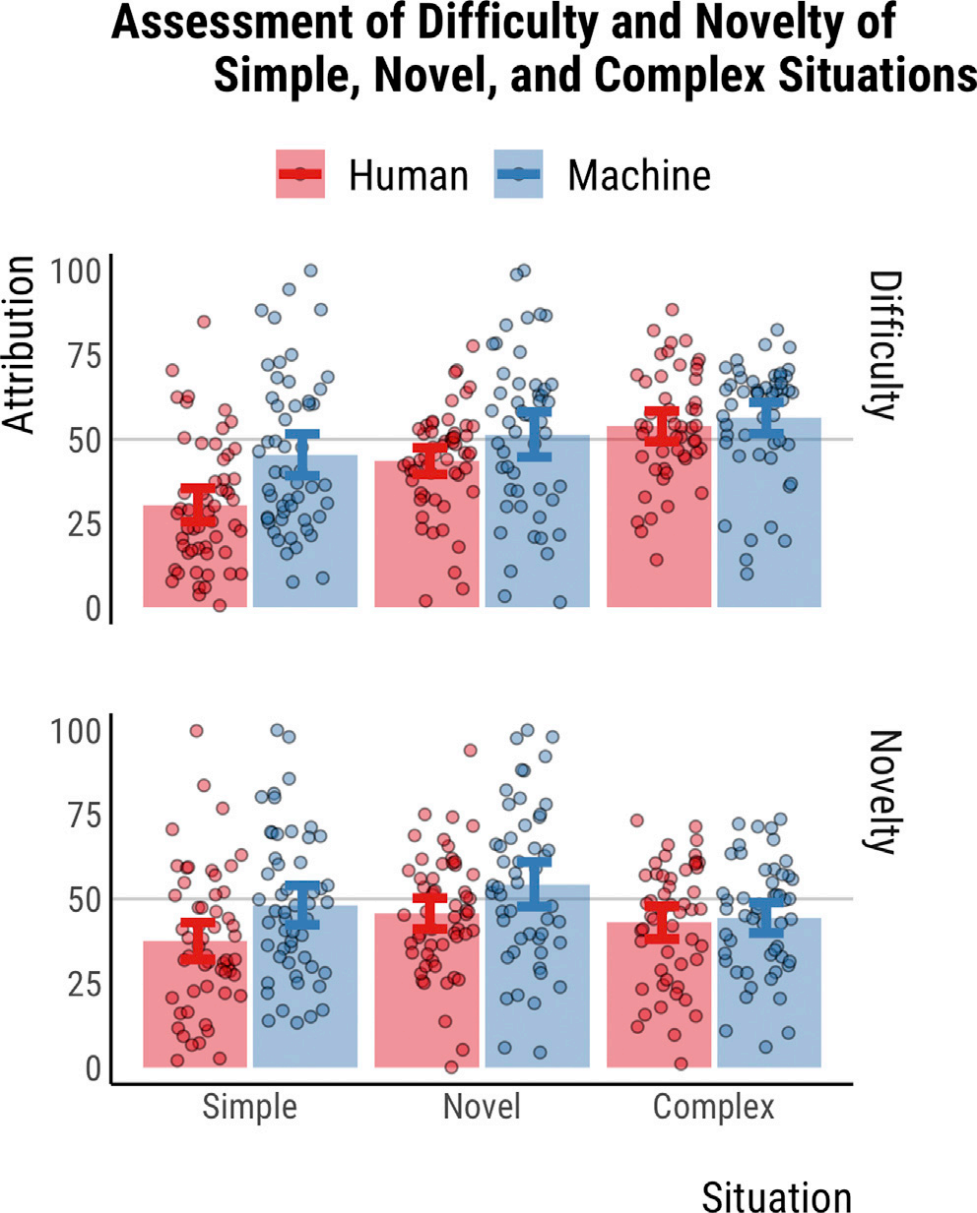
Assessment of difficulty and novelty of simple, novel, and complex situations in study 2 Data from study 2 (n = 317). Participants were randomly allocated to one of six groups. The x axis represents the situation difficulty (simple vs. novel vs. complex).The y axis represents difficulty or novelty assessment. Blue and red bars represent the mean assessment to the described scenarios featuring a machine driver (AV) and a human driver, respectively. Error bars represent 95% confidence intervals of the mean. Each circle represents an individual's difficulty or novelty scores (averaged over five stories). For situations featuring a human driver, the mean difficulty score in complex situations is significantly higher than in novel [differences in means = −51.46, 95% CI: [-89.89, −13.03], p = .005] and simple [differences in means = −116.65, 95% CI: [-155.24, −78.05], p < .001] situations. For situations featuring a machine driver, the mean difficulty score in complex situations is significantly higher than in simple situations [differences in means = −54.61, 95% CI: [-104.34, −4.88], p = .028], and the mean novelty score in novel situations is significantly higher than in complex situations [differences in means = 49.75, 95% CI: [.92, 98.58], p = .045]. Data are represented as mean ± SEM.

To test for participants' perception of how difficult human and machine drivers would find particular situations, and to examine the success of the experimental manipulation of the study's items, we observed people's ratings of situations' difficulty and novelty. Specifically, the five separate ratings that were given by participants for each item was summed into a novelty and difficulty score. The descriptive statistics (see Figure 4) imply that the items elicited the desired response, with items describing novel situations having the highest novelty scores, and items describing complex situations having the highest difficulty scores.

For novelty scores, results from a 2 × 3 ANOVA (driver x situation) show that identical driving situations are scored as more novel if they have a machine driver in them rather than a human driver [F(314,3) = 9.65, p = .002] and that different driving situations produce a significant difference in novelty scores [F(314,3) = 4.15, p = .017]. Post hoc comparisons using the Tukey HSD test indicated that the mean novelty score in novel situations (M = 248.68, SD = 105.35) was significantly higher than in simple situations (M = 213.62, SD = 108.63).

For difficulty scores, results from a 2 × 3 ANOVA (driver x situation) show that identical driving situations are scored as more difficult if they have a machine driver in them rather than a human driver [F(314,3) = 14.98, p < .001] and that different driving situations produce a significant difference in difficulty scores [F(314,3) = 20.99, p < .001]. Post hoc comparisons using the Tukey HSD test indicated that the mean difficulty score in complex situations (M = 275.3, SD = 83.05) was significantly higher than in simple (M = 188.93, SD = 110.95) and novel situations (M = 236.17, SD = 100.17). Further, the mean difficulty score in novel situations was significantly higher than in simple situations.

Further, we examined whether trust in other drivers or AVs were predictive of blame judgments and causal attributions. For this, four separate linear regressions were conducted – two for participants in machine driving scenarios and another two for participants in human driving scenarios. For groups with human drivers, trust in other drivers did not significantly predict blame judgments and causal attributions. For groups with machine drivers, the results of two linear regressions showed that trust in AVs significantly predicted blame judgment [F(153, 2) = 4.06, p = .046, R^2^ = .026] and causal attribution [F(153, 2) = 5.19, p = .024, R^2^ = .033]. The results show that people's trust in AVs predicts their judgments and attributions of AVs.

Finally, we examined participants' expectations of whether they thought a mistake was going to happen or not after completing the items (human: M = 5.02, SD = 1.45; machine: M = 5.35, SD = 1.25). The results of an independent sample T test show that people's expectations for a mistake were significantly larger for machines than humans [d(315,2) = -.24, p = .017]. Expectations were then used to predict participants' judgments and attributions, using two separate linear regressions. When using blame attribution as the dependent variable, the linear regression produced a significant effect [F(315,2) = 4.63, p = .032, R^2^ = .014]. This finding was replicated when causality attribution was the dependent variable [F(315,2) = 9.09, p = .003, R^2^ = .028]. The results show that people's expectations predict their judgments and attributions.

We reviewed participants' qualitative responses to how they made their choices. The three main themes were that participants' own experience with driving, the situation they were analyzing, and that the driver they were judging informed their decisions.

## Discussion

Before being deployed on the road, AVs need to reach a satisfactory level of competence. What is satisfactory, however, may be measured against satisfactory levels of human driving. But comparisons between humans and machines are hard to make, since each is challenged differently, even when facing the same set of situations. While AVs operating based on state-of-the-art technology can navigate fast-calculation, quick-reaction types of situations better than humans, they still struggle with novel (yet seemingly simple from a human perspective) situations. Because of this, one would expect that people will blame machine drivers less than human drivers for making mistakes in novel situations but blame them more in complex situations that rely on handling more information or calculation.

Surprisingly, we find that this is not the case. In two studies, we find that participants blamed machine drivers more than human drivers for mistakes in both complex and novel situations. The differences in blame toward machines and humans may seem small on a scale 1-100 (meaning only few more people would find machines more blameworthy than humans, than vice versa). However, such differences are likely to map to practical turning points in real life. Our studies are done with a group of independent individuals faced with neutral description of scenarios. This ignores two factors: (1) media influence and (2) social influence, both of which are expected to magnify the difference. As for (1), If our sample is any representation of journalists, this will be reflected in more blame-the-machine biased articles, that are read by many more individuals. As for (2), it is plausible to assume that social influence of judgment happens according to a majority-voting model (majority of your neighbors determine your “state” with high probability p_agree_) (Campos et al., 2003; Liggett, 2012), and that influence propagates over our social networks, represented as “small-world” networks (Watts and Strogatz, 1998; Amaral et al., 2000). In such settings, “who is blamed more?” matters more than “how much blamed more?” in shaping the final collective judgment (Ray et al., 2021).

There are multiple possible explanations that may help understand these findings. The first possibility is that people were not sensitive to the difficulty or novelty of these situations in a way that follows the desired experimental manipulations. However, this is unlikely, given that (as illustrated in Figure 4) participants assigned higher novelty scores for novel situations than for simple or complex situations when faced by machines. They also assigned higher difficulty scores for complex situations than the other two when faced by human drivers. Finally, novel and complex situations were considered more difficult than simple situations when faced by machine drivers.

One factor that can influence blame attribution is whether responsibility is shared. Previous work showed that people factor in the role of other agents in the broader system when making judgments (Lagnado et al., 2013). Given this, if a machine and a human perform an identical action with the same consequence, people might view them differently in terms of their causality and blameworthiness due to the other agents that are somewhat responsible for their behavior. However, this also seems like an unlikely explanation for our findings. Compared to humans, one would expect that machines should be “sharing” the blame with more agents, such as developers, designers, data scientists, data sets and manufacturers, a problem that has been identified before as “AI Responsibility Gap” (Matthias, 2004), and “moral crumple zone” (Elish, 2019).

One possible explanation is that participants perceive machines to be more competent at driving than humans. Expectations of someone's skills influence people's blame judgment for an outcome (Gerstenberg et al., n.d.). Specifically, when one has a high prior expectation of how someone will behave, they will see them as more blameworthy if they underperform and cause a negative outcome. However, this explanation is refuted by our post-assessment questions which found participants expressing higher likelihood of making a mistake (in the considered situations) for machine drivers than for human drivers. This runs counter to the literature on the public's risk acceptance of AVs, which shows that the public expects AVs to be significantly safer than human drivers, feel reasonably safe riding in an AV, and would allow AVs on public roads (Nees, 2019). Our participants' expectation of failure can be seen as an expectation of a driver's ability to avoid a mistake i.e., higher perceived competence at driving for humans.

Another possible explanation is that participants hold machines to higher standards than humans for the task of driving. In this case, even when people perceive machines as less competent drivers than humans, they still want them to follow higher standards before they are allowed to drive us. Our finding that for AVs, higher trust predicts higher blame may somewhat support this conclusion. Given that we do not have data that refutes or confirms this possibility, this remains as a possible explanation for why machines receive more blame than humans.

Finally, the answer may lie in the attribution of causal responsibility. People judge agents as more blameworthy for an outcome if they see them as more causally responsible for that outcome (Gerstenberg and Lagnado, 2010). In this case, if machines are seen as more causally responsible for the described mistakes, we would expect them to blame them more for these mistakes. Our data show that causal attribution results mirror the blame attributions for all conditions. However, this does not provide a satisfactory explanation. It only shifts the focus to causal responsibility: Why do people find machines more causally responsible than humans irrespective of the difficulty of the situation?

The immediate future is likely to see machines assuming new instrumental roles in industry and governance. This has, and will, lead to new situations where the developers of such intelligent machines are not able to fully predict their machines behavior and thus mistakes. The current study contributes to the existing literature (Malle et al., 2015; Awad et al., 2020; Bennett et al., 2020; Hidalgo et al., 2021) seeking to understand how situations of failure shape the public's attitude toward machines. Exploring how this is likely to unfold is a crucial step forward toward realizing the potential benefit of this technology.

### Limitations of the study

The study has three limitations pertaining to its participants, measures and explanations it draws from its results. First, although our sample size met the requirements of a power analysis (see Participants section in Methods), a larger sample size across the two studies would have made for more conclusive results. Further, as our sample was recruited from MTurk and included participants from the UK, it was not fully representative. Second, the measure used for people's expectations of whether a mistake was about to happen was ad hoc, thus participants' expectations could have been influenced by the experimental items which described the driving situations. Finally, the current study cannot fully provide a definitive explanation for why machines are blamed more across all driving situations.

### Resource availability

#### Lead contact

Further information and requests for experimental materials and data should be directed to and will be fulfilled by the lead contact, Matija Franklin (matija.franklin@ucl.ac.uk).

#### Materials availability

All items used in the online experiment are available from the lead contact without restriction.

#### Data and code availability

All data generated or analyzed during this study are currently available in the Figshare repository. The data from study 1 are available here https://figshare.com/articles/dataset/Blaming_Automated_Vehicles_Study_1_/12982085. The data from study 2 are available here https://figshare.com/articles/dataset/Blaming_Automated_Vehicles_Study_2_/12982103.

## Methods

All methods can be found in the accompanying Transparent methods supplemental file.

## Supplemental information

Document S1. Transparent methods and Table S1
