# Supplementary material for: Blaming automated vehicles in difficult situations
Source: iScience. 2021 Mar 1;24(4):102252. doi: 10.1016/j.isci.2021.102252 (PMC7995526; doi:10.1016/j.isci.2021.102252)
Supplement: Document S1. Transparent methods and Table S1 [file mmc1.pdf]

iScience, Volume *24*

Supplemental information

Blaming automated vehicles in difficult situations

Matija Franklin, Edmond Awad, and David Lagnado

<u>**Supplemental Data Items**</u>

Item Number	F Causality	F Blame
1	.634	.005
2	11.603***	18.881***
3	2.154	.791
4	7.375**	4.335*
5	.034	.017

Table S1. **Results from individual item ANOVAS between human and machine groups in Study 2, related to Figure 3**. Ten separate ANOVAs which explored the differences in mean causality or blame between groups judging machine or human drivers, for the five different items. For all significant scores, machine drivers received significantly more blame judgments or causal attributions than human drivers $p< .05$ = *, $p< .01$ = **, $p< .001$ = ***]. For non-significant scores in the remaining three items, the same direction either held (i.e., machines were attributed more blame or causality) or the differences were negligible.

<u>Transparent Methods</u>

Study 1

Design
The study used a between-subject design with four experimental groups, which differed in terms of the drivers and mistakes people were judging - human drivers making mistakes in simple situations, AI drivers making mistakes in simple situations, human drivers making mistakes in complex situations, AI drivers making mistakes in complex situations. All groups only differed in the traffic accident items it showed to participants, while all groups showed the same instructions and asked the same additional questions about self-driving car knowledge, and sociodemographic questions. Participants were randomly assigned to the four experimental groups. In response to each item, participants made causal attributions, blamed judgments and were asked whether or not they would make the same mistake, and would someone they know have made the same mistake. The summary of the participants' attributions and judgments - 'Total Blame' and 'Total Cause' - were the study's main dependent variables. The sum of people's response to whether they would make the mistake, or know someone who made a mistake, were used as one of the study's predictor variables.

For each item, participants were additionally asked to write why they made the judgment they made, and also what the driver could have done differently. These qualitative questions served as a way of collecting information that could inform or inspire the design of future studies.

Experimental Procedure
Participants were told that they were going to be presented with six different traffic accident scenarios, for which they were going to make judgments of causality and blame, as well as some additional questions. Participants had the right to leave the study at any point, but responding to all items was mandatory. Informed consent was obtained from all participants before the beginning of the study.

Participants were first shown instructions for what they were going to do in the study. They were then split into one of the four groups. They then responded to the six traffic accident scenarios. Finally, participants were asked what they knew about self-driving cars, and answered six sociodemographic questions. This item order was used because the sociodemographic questions could prime participants' judgments and attributions, an effect known as social priming. Study data will be publicly available on GitHub.

Participants
A power analysis was conducted in order to determine the smallest sample size suitable to detect the effects of an ANOVA. The alpha level set to 0.05 and a power set to 0.8. The estimation indicated that the minimum number of participants had to be 180, with a final sample of 198 achieved.

For the initial study, participants had to be from the UK and above the age of 18. The 198 participants (53% females; median age of 27) were recruited via Prolific.

Measures
Traffic Accident Scenarios
The six scenarios were constructed so that mistakes in complex situations were harder to avoid than mistakes in simple situations. The mistakes always resulted in a crash. Across the four experimental groups the traffic accident scenarios were mostly similar, but differed in two crucial ways. First, for AI groups, the driver was referred to as the “self-driving car”, while for human groups, the driver was referred to as the “person” or “driver”. Second, there was always one sentence per scenario that differed between simple and complex mistake scenarios. See the Supplementary Information for all items.

Judgments and Attributions
To measure the causal attributions and blame judgments, participants were asked "To what extent did the X cause the crash?" and "To what extent is the X to blame for the crash?", respectively, with X referring to either the human driver or self-driving car. These were made on a scale of 0-100. Participants were also asked “Would have you made the same mistake?” and “Would someone you know have made the same mistake?” to which they could respond yes or nor. For all of the described

questions, participants were asked once per accident item, with six items in total per experimental group.

Qualitative
For every traffic accident scenario, participants were asked the two following qualitative questions - “Why did you make this judgment?” and “What could the X have done differently?” with X referring to either the human driver or self-driving car.

Sociodemographic
Participants were asked to provide information about six sociodemographic factors - age, education level, gender, household income, political views and religious views.

Other
Participants were asked - “What do you know about self-driving cars?”.

Experimental Items

*Human driver experimental groups referred to the driver as “person” and machine driver experimental groups referred to the driver as “self-driving car”. Below are the items for the human driver experimental groups.

I Simple Driving Situations
a. Items
1. A person is driving on a highway. There is another car driving in front. The car in front brakes unexpectedly and the person crashes into it.
2. A car driver is about to enter a multi-lane roundabout. The roundabout is empty. The driver enters and crashes into the centre of it.
3. An ambulance has its sirens on and is trying to get through traffic. The traffic is very light, and a driver in traffic only needs to move to the side to make space. The driver tries to do that but hits a signpost and causes a traffic jam. As a result, the ambulance was too late to save the patient.
4. A person is driving down a quiet road, abiding by the speed limit. Twenty meters ahead of him a pedestrian jumps on the road. The person makes a very sudden turn and crashes into a wall on the side of the road.
5. A person is driving on a highway. There is another car driving in front. The car in front brakes unexpectedly and the person crashes into it.
6. A person is driving down a quiet road, abiding by the speed limit. Twenty meters ahead of him, on the side of the road he sees a person who is about to cross the road. The driver keeps driving and crashes into the person on the road.

b. Questions (repeated for all items)
1. To what extent did the driver cause the crash? 0-100 (slider)
2. To what extent is the driver to blame for the crash? 0-100 (slider)
3. Would have you made the same mistake? i. Yes ii. No
4. Would someone you know have made the same mistake? i. Yes ii. No
5. Why did you make this judgment?
6. What could the driver have done differently?

II Complex Driving Situations
a. Items
1. A person is driving on a highway, it’s raining and the visibility is bad. There is another car driving in front. The car in front brakes unexpectedly and the person crashes into it.
2. A car driver is about to enter a multi-lane roundabout. The roundabout is very busy. The driver enters and crashes into the centre of it.
3. An ambulance has its sirens on and is trying to get through traffic. The traffic is very busy, and a driver in traffic needs to do some manoeuvring in order to make space. The driver tries to do that but he hits a signpost and causes a traffic jam. As a result, the ambulance was too late to save the patient.

4. A person is driving down a quiet road, abiding by the speed limit. Two meters ahead of him a pedestrian jumps on the road. The person makes a very sudden turn and crashes into a wall on the side of the road.
5. A person is driving down the road and the car in front suddenly stops. The person crashes into the car.
6. A person is driving down a quiet road, abiding by the speed limit. Twenty meters ahead of him, on the side of the road he sees an ostrich that is about to cross the road. The driver keeps driving and crashes into the ostrich on the road.

b. Questions (repeated for all items)
*Same as for other experimental groups (See I Simple Driving Situations, b. Questions)

III Additional and Demographic Questions
1. What do you know about self-driving cars?
2. What is your year of birth?
3. What is the highest level of school you have completed or the highest degree you have received?
4. What is your sex?
5. Information about income is very important to understand. Would you please give your best guess? Please indicate the answer that includes your entire household income in (previous year) before taxes.
6. Please indicate your political views from extremely progressive (left) to extremely conservative (right). Where would you place yourself on this scale?
7. Please indicate your religious views from extremely non-religious (left) to extremely religious (right). Where would you place yourself on this scale?

Study 2
Design
The study used a between-subject design with six experimental groups, which differed in terms of the drivers and mistakes people were judging - human drivers making mistakes in simple situations, AI drivers making mistakes in simple situations, human drivers making mistakes in complex situations, AI drivers making mistakes in complex situations, human drivers making mistakes in novel situations, AI drivers making mistakes in novel situations. As with Study 1, all groups only differed in the traffic accident items it showed to participants, while all groups showed the same instructions and asked the same additional (described in Experimental Procedure and Materials) and sociodemographic questions. Participants were randomly assigned to the six experimental groups. In response to each item, participants made causal attributions, blamed judgments and were asked to judge to what extent the described driving situation was difficult and novel. The summary of the participants' attributions and judgments - 'Total Blame' and 'Total Cause' - were the study's main dependent variables. Participants summed judgments on item difficulty and novelty were used to validate the items.

Experimental Procedure
Participants were told that they were going to be presented with five different traffic accident scenarios, for which they were going to make judgments of causality and blame, as well as some additional questions. Participants had the right to leave the study at any point, but responding to all items was mandatory. Informed consent was obtained from all participants before the beginning of the study.

Participants were first shown instructions for what they were going to do in the study. They were then split into one of the six groups. They then responded to the five traffic accident scenarios. The order in which the five items were presented was randomised. The order of these questions for each individual item was also randomised. Participants were then asked whether they wanted to buy a car or self-driving car, and whether they trust other drivers or self-driving cars, depending whether they were in a group that judged human or AI drivers, respectively. They were also asked whether or not they knew a mistake was about to happen in the previous items that described traffic accident scenarios. Finally, to validate one of the experimental items, the participants were asked whether they (or anyone they know) have ever encountered a deer or an ostrich on the road, depending on the experimental group they were in (people judging novel mistakes were asked about the ostrich, while others were asked about the deer).

Participants were then asked some additional questions. First, they were asked to write why they made the previous choices while evaluating the traffic accident items. They were then asked whether they have a driving licence. If they replied yes, they were then asked whether or not they own a car and how many times do they drive per week, on average. Finally, they were asked the same six socio-demographic questions from Study 1. As with Study 1, the item order was used because the sociodemographic questions could prime participants' judgments and attributions. Study data will be publicly available on GitHub.

Participants

A power analysis was conducted in order to determine the smallest sample size suitable to detect the effects of an ANOVA. The alpha level set to 0.05 and a power set to 0.8. The estimation indicated that the minimum number of participants had to be 216, with a final sample of 317 achieved.

For the initial study, participants had to be from the UK and above the age of 18. The 317 participants (36% females; median age of 33) were recruited via MTurk.

Measures

Traffic Accident Scenarios

The five scenarios were constructed so that mistakes in novel and complex situations were harder to avoid than mistakes in simple situations. Two mistakes resulted in a crash, another two in the passengers being late and one resulted in a car crash. Across the six experimental groups the traffic accident scenarios were mostly similar, but differed in two crucial ways. First, for AI groups, the driver was referred to as the "self-driving car", while for human groups, the driver was referred to as the "person" or "driver". Second, there was always one or two sentences per scenario that differed between simple, complex and novel mistake scenarios. See the Supplementary Information for all items.

Judgments and Attributions

To measure the causal attributions and blame judgments, participants were asked "To what extent did the X cause the Y?" and "To what extent is the X to blame for the Y?", respectively, with X referring to either the human driver or self-driving car, and Y referring to the outcome of the mistake. These were made on a scale of 0-100. Participants were also asked "To what extent is the described driving situation novel?" and "To what extent is the described driving situation difficult?" to which they could respond on a 0-100 scale. For all of the described questions, participants were asked once per accident item, with five items in total per experimental group.

Qualitative

After responding to the traffic accident scenarios, participants were asked "Please describe how you made your choices in this HIT".

Sociodemographic

As with Study 1, participants were asked to provide information about six sociodemographic factors - age, education level, gender, household income, political views and religious views.

Other

Participants in human driver experimental groups were asked to reply to "I want to buy a car" and "I trust other drivers" on a 1-7 scale (Strongly disagree - Strongly agree). Participants in AI driver experimental groups were asked to reply to "I want to buy a self-driving car one day" and "I trust self-driving cars" on a 1-7 scale (Strongly disagree - Strongly agree).

Participants in all groups were asked to reply to "In most of the previous stories, as I was reading the story, I knew that a mistake was about to happen" on a 1-7 scale (Strongly disagree - Strongly agree).

Participants in simple and complex mistake experimental groups were asked "Have you, or anyone you know, ever encountered a deer on the road?" to which they could reply "Yes" or "No". Participants in the novel mistake experimental groups were asked "Have you, or anyone you know, ever encountered an ostrich on the road?" to which they could reply "Yes" or "No".

Finally, Participants were asked "Do you have a driving licence?" to which they could reply "Yes" or "No". If they replied with "Yes", they were additionally asked "Do you own a car" to which they could reply "Yes" or "No", as well as "On average, how many times a week do you drive?" to which they needed to respond with a number.

Experimental Items

I Simple Driving Situations

a. Items

1. A person is driving down a road. There is a STOP sign that is clearly visible. The driver doesn’t stop and crashes into another car that had priority at that crossing.
2. A person is driving down a quiet road, abiding by the speed limit. Twenty meters ahead of him/her, on the side of the road, he/she sees a deer that is about to cross the road. The driver turns suddenly and crashes into a lamppost.
3. A person driving a car is stopped at a red traffic light. The traffic light turns green. The driver does not move and he/she causes a traffic jam.
4. A person is driving down a two way road. There is a passenger in the back seat. The driver approaches another car that is broken down. There is room for the driver to overtake the broken down car and it is clearly legal to overtake on that part of the road. The driver stops driving and shuts down at a safe distance from the broken down car. The passenger in the car is late for a meeting.
5. A person is driving towards the airport. There is a passenger in the back seat. There is little traffic along the route. This makes it easy for the driver to change to a faster route. However, the driver does not change routes and as a result the passenger is late for his/her flight.

b. Questions (repeated for all items)

*The questions below refer to the questions participants got in response to items that described a crash. The text would change accordingly if the item was referring to a traffic jam or a passenger being late

1. To what extent did the driver cause the crash?
2. To what extent is the driver to blame for the crash?
3. To what extent is the described driving situation novel?
4. To what extent is the described driving situation difficult?

c. Questions (standalone and asked after the five items)

1. I want to buy a car. 1-7 (Strongly Disagree-Strongly Agree)
2. I trust other drivers. 1-7 (Strongly Disagree-Strongly Agree)
3. In most of the previous stories, as I was reading the story, I knew that a mistake was about to happen. 1-7 (Strongly Disagree-Strongly Agree)
4. Have you, or anyone you know, ever encountered a deer on the road? i. Yes ii. No

II Novel Driving Situations

a. Items

1. A person is driving down a road. There is a STOP sign but the S has been scratched off and now the sign reads TOP. The driver doesn’t stop and crashes into another car that had priority at that crossing.
2. A person is driving down a quiet road, abiding by the speed limit. Twenty meters ahead of him/her, on the side of the road, he/she sees an ostrich that is about to cross the road. The driver turns suddenly and crashes into a lamppost.
3. A person driving a car is stopped at a red traffic light. The traffic light turns green, but the green glass is broken so the light appears white. The driver does not move and he/she causes a traffic jam.
4. A person is driving down a two way road. There is a passenger in the back seat. The driver approaches another car that is broken down. There is room for the driver to overtake the broken down car but the double white line indicates that it is illegal to overtake on that part of

the road. The driver stops driving and shuts down at a safe distance from the broken down car. The passenger is late for a meeting.

5. A person is driving towards the airport. There is a passenger in the back seat. The radio announces that the concert in a nearby stadium has finished earlier than planned and there will be a lot of traffic along the route. This will soon make it tricky for the driver to change to a faster route. However, the driver does not change routes and as a result the passenger is late for his/her flight.

b. Questions (repeated for all items)
*Same as for other experimental groups (See I Simple Driving Situations, b. Questions)

c. Questions (standalone and asked after the five items)
*Same as for other experimental groups (See I Simple Driving Situations, c. Questions) with one exception - question 4:

1. Have you, or anyone you know, ever encountered a ostrich on the road? i. Yes ii. No

III Complex Driving Situations

a. Items

1. A person is driving down a road. There is a STOP sign but it is foggy and the visibility is very bad. The driver doesn't stop and crashes into another car that had priority at that crossing.
2. A person is driving down a quiet road, abiding by the speed limit. Two meters ahead of him/her, on the side of the road, he/she sees a deer that is about to cross the road. The driver turns suddenly and crashes into a lamppost.
3. A person driving a car is stopped at a red traffic light. The traffic light turns green, but only for one second due to a fault with the traffic light. The driver does not move and he/she causes a traffic jam.
4. A person is driving down a two way road. There is a passenger in the back seat. The driver approaches another car that is broken down. There is little room for the driver to overtake the broken down car with incoming traffic but it is clearly legal to overtake on that part of the road. The driver stops driving and shuts down at a safe distance from the broken down car. The passenger is late for a meeting.
5. A person is driving towards the airport. There is a passenger in the back seat. It is rush hour and there is a lot of traffic along the route. This makes it tricky for the driver to change to a faster route. However, the driver does not change routes and as a result the passenger is late for his/her flight.

b. Questions (repeated for all items)
*Same as for other experimental groups (See I Simple Driving Situations, b. Questions)

c. Questions (standalone and asked after the five items)
*Same as for other experimental groups (See I Simple Driving Situations, c. Questions)

IV Additional and Demographic Questions

1. Please describe how you made your choices in this HIT.
2. Do you have a driving licence? i. Yes ii. No [If "Yes" is Selected - 1. Do you own a Car? i. Yes ii. No; On average, how many times a week do you drive?]
3. What is your year of birth?
4. What is the highest level of school you have completed or the highest degree you have received?
5. What is your sex?
6. Information about income is very important to understand. Would you please give your best guess? Please indicate the answer that includes your entire household income in (previous year) before taxes.
7. Please indicate your political views from extremely progressive (left) to extremely conservative (right). Where would you place yourself on this scale?
8. Please indicate your religious views from extremely non-religious (left) to extremely religious (right). Where would you place yourself on this scale?
